# MiR-200c-3p expression may be associated with worsening of the clinical course of patients with COVID-19

**DOI:** 10.22099/mbrc.2021.40555.1631

**Published:** 2021-09

**Authors:** Ruan Pimenta, Nayara I. Viana, Gabriel A. dos Santos, Patrícia Candido, Vanessa R. Guimarães, Poliana Romão, Iran A. Silva, Juliana A. de Camargo, Diná M. Hatanaka, Paula G. S. Queiroz, Alexandre Teruya, Leandro Echenique, Bruno A. M. P. Besen, Katia R. M. Leite, Victor Srougi, Miguel Srougi, Sabrina T. Reis

**Affiliations:** 1 Laboratory of Medical Investigation (LIM55), Urology Department, Faculdade de Medicina da Universidade de São Paulo (FMUSP), São Paulo, Brazil; 2 Moriah Hospital, São Paulo, SP, Brazil; 3 Instituto Central, Hospital das Clinicas HCFMUSP, Faculdade de Medicina, Universidade de SaoPaulo, Sao Paulo, SP, Brazil

**Keywords:** MicroRNA, COVID-19, SARs-CoV-2, Biomarkers

## Abstract

COVID-19 represents a public health emergency, whose mechanism of which is not fully understood. It is speculated that microRNAs may play a crucial role in host cells after infection by SARS-CoV-2. Thus, our study aimed to analyze the expression of miR-200c-3p in saliva samples from patients with COVID-19. One handred eleven samples from patients with COVID-19 were divided into 4 groups. Group I: 39 patients negative for Covid-19; Group II: 37 positive and symptomatic patients, with no indication of hospitalization; Group III: 21 patients with respiratory disorders (hospitalized); Group IV: 14 patients with severe conditions (oxygen therapy). The expression levels of miR-200c-3p were determined using qPCR. We found greater expression of miR-200c-3p in patients in group IV (*p*<0.0001), and also verified that patients aged ≥42 years had a higher expression of this miR (*p*=0.013). Logistic regression analysis revealed that the expression of miR-200c-3p and systemic arterial hypertension are factors independently associated with patients in group IV (*p*<0.0001). Our results suggest that miR-200c-3p is a predictor of severity independent of COVID-19 risk factors, which could represent a way of screening patients affected by SARS-CoV-2.

## INTRODUCTION

MicroRNAs (miRNAs) are small endogenous molecules that do not encode proteins that bind mRNA targets of several genes and can control their expression at the posttranscriptional level [1]. miRNAs have peculiarities in several biological pathways, in addition to important functions in a wide variety of pathological conditions, such as viral infections [2], where they act in the regulation of the host response [3].

Viral infections regulated by the action of miRNA have already been described in several hosts, both in infections caused by DNA and RNA viruses [4]. With the efforts of several studies, significant progress has recently been made in understanding the molecular mechanisms underlying respiratory virus infections and their interaction with the host. The identification and characterization of the expression profile of microRNAs after respiratory viral infections and their implications for the infection pathway is an important tool to understand the virus-host interaction and several other mechanisms [5]. Although the mechanism of COVID-19 infection is not fully elucidated, it is speculated that microRNAs may play a crucial role in host cells after SARS-COV-2 infection [6, 7].

COVID-19 (Coronavirus Disease-2019) represents a public health emergency [8]. Worldwide, there are 195 more affected countries, with approximately 19.5 million confirmed cases and 728,000 deaths [9]. Among those infected, approximately 15% progress to the most severe form of the disease, and the mortality rate is 2.3% [10]. Patients with hypertension and/or diabetes are at increased risk of progression COVID-19 [11]. In addition to these comorbidities, age also appears to be among the risk factors of COVID-19, where the middle-aged population and the elderly have the highest mortality rates [10]. Recently, clinical and epidemiological investigations have shown that the gender gap may be related to patients affected by COVID-19, where men have more severe forms and a higher lethality rate when compared to women [12]; however, this mechanism is not fully understood.

Studies with microRNAs can contribute not only to the understanding of the virus-host interaction but also to stratify the different degrees of severity presented by COVID-19. In this sense, miR-200c-3p, which is associated with viral infections, including influenza A [13], presents itself as a candidate microRNA for investigation at COVID-19. The analysis of its expression in groups of patients who exhibit different degrees of aggressiveness of the disease could contribute to better screening of patients affected by SARS-CoV-2. Thus, the present study aimed to analyze the expression of miR-200c-3p in saliva samples from patients diagnosed with COVID-19.

## MATERIALS AND METHODS

**Patients: **This quantitative longitudinal analytical study was carried out in two hospitals in the city of São Paulo - Hospital Moriah and Hospital das Clinicas of the Faculty of Medicine of the University of São Paulo (HCFMUSP), Brazil from June to October 2020. The sample consisted of 111 patients who donated saliva samples. All patients were tested for SARs-CoV-2 using the qPCR (nasopharyngeal swab) technique. Patients were divided into 4 groups. Group I was composed of 39 patients with a negative test for Covid-19. Group II was composed of 37 patients with symptomatic clinical conditions and with no indication of hospitalization (absence of respiratory dysfunction). Group III was composed of 21 symptomatic patients who had respiratory disorders; and therefore, these patients needed hospitalization. Group IV, consisted of 14 patients with severe conditions, requiring oxygen therapy and, consequently, were admitted to the Intensive Care Unit of HCFMUSP. Clinical-demographic characteristics of patients are shown in Table 1. This study was approved by the Research Ethics Committee of the Faculty of Medicine of the University of São Paulo (4,113,149). All patients agreed to participate in the study and signed an informed consent form.

**Procedures: **To measure the levels of microRNA expression, all patients were instructed to produce a saliva sample at the time of the COVID-19 test collection. The patients remained for at least 1 minute without swallowing and at the end, they spit all the saliva stored in the mouth in the collection tube. In patients admitted to the ICU on oxygen therapy, a sublingual smear was performed with the aid of a swab and subsequently, it was immersed in a collecting tube containing 2 mL of saline solution. All collections were guided and supervised by the nursing team. The samples were stored at -20°C until extractions were performed. 

**Table 1:** Clinical-demographic characteristics of patient

** Age (years)**					
**Variation**	**Min. 19**		**Max. 77**	**Average 42**	
	<42 years		≥42 years	*p*	
**Mean** **(Std. Deviation)**	32.24 (6.532)		54.06 (9.063)	<0.0001	
**Male**	n= 60			54.05 %	
**Feminine**	n= 51			45.95 %	
		
**% (n)**	**I**	**II**	**III**	**IV**	***p***
**Hypertension**	10.26 (4)	11.11 (4)	25.00 (5)	71,43 (10)	<0.0001
**Diabetes**	0.00 (0)	2.78 (1)	25.0 (5)	42.86 (6)	<0.0001
**Hypertension And Diabetes**	0.00 (0)	0.00 (0)	19,05 (4)	35,71 (5)	<0.0001

**Extraction of microRNA and quantitative real-time polymerase chain reaction: **To extract the total RNAs, the EasyExtract DNA-RNA kit (Interprise^®^) was used, following the manufacturer's recommendations. After extraction, the miRNA cDNA was generated using the TaqMan miRNA reverse transcription kit (Applied Biosystems, Foster City, CA). The reactions were incubated at 16ºC for 30 min, 42ºC for 30 min, and 85ºC for 30 min. The expression levels of hsa-miR-200c-3p (478351_mir) were analyzed by qPCR using the ABI 7500 Fast Real-Time PCR System (Applied Biosystems). The target sequence was amplified in a 10 µL reaction mixture containing 2 µL of HOT FIREPol Probe Universal qPCR Mix (Solis BioDyne), 0.5 µL of TaqMan miRNA for miR-200c, 1 µL of cDNA, and 6.5 µL of DNase-free water. The conditions of the PCR cycles were 2 min at 50ºC, 10 min at 95ºC and then 45 cycles of 15 s at 95ºC, and 1 min at 60ºC. The normalized relative expression was calculated by thresholding the Ct values of miR-200c with the Ct value of miR-191 (calibrator) [14]. The data were analyzed in DataAssist Software (Applied Biosystems, USA). All qPCR reactions were performed in duplicate.

**Statistical analysis: **We used the Kolmogorov-Smirnov test to assess the normality of our data. When the data were parametric, we use Student's *t*-test to compare the levels of expression between groups. When the data were nonparametric we use the Mann-Whitney test to compare the levels of expression between groups. To analyze more than two groups we use the ordinary one-way ANOVA test for the hypothesis test. Contingency analysis was performed using the Chi-squared test. To assess independently associated correlations between group IV and the miR-200c, we performed a logistic regression. The graphs and the statistical analysis were performed using GraphPad Prism 8 software. The result was considered significant when *p*≤0.05. The data are detailed in Supplementary 1, 2, and 3. 

## RESULTS

**Figure 1:**
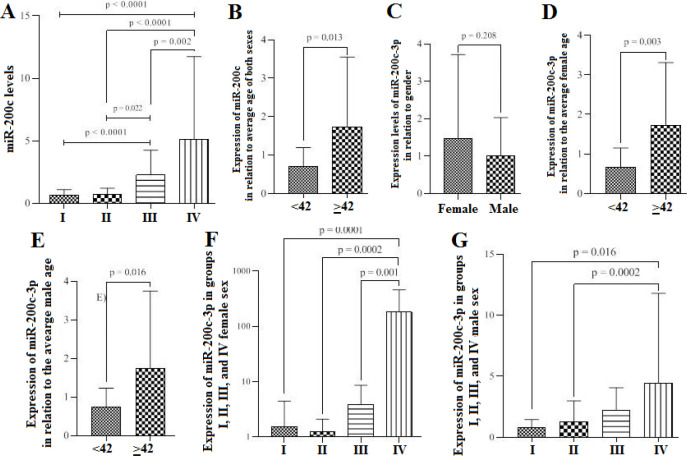
Expression levels of miR-200c-3p in the different stages of COVID-19. A) Differential expression of miR-200c-3p in groups I, II, III and IV. B) Expression in relation to the average age of both sexes of patients with COVID-19. C) Expression levels of miR-200c-3p in relation to genders. D) Expression of miR-200c-3p in relation to the average age considering only the female sex. E) Expression of miR-200c-3p in relation to the average age considering only the male sex. F) Expression of miR-200c-3p in groups I, II, III and IV considering only the female sex. G) Expression of miR-200c-3p in groups I, II, III and IV considering only the male sex

When analyzing the expression of miR-200c-3p, we observed that there was an increased expression pattern according to the severity of the patients, with group IV showing a higher mean of expression when compared to the other groups (5,147 ± 6,601, *p*<0.0001) (Fig. 1A). It was shown that patients aged ≥42 years old have higher levels of expression of miR-200c-3p (0.711 ± 0.482 and 1,737 ± 1,819, *p*=0.013) (Fig. 1B). Regarding gender, there was no significant difference between women (45.95%) and men (54.05%), *p*=0.208 (Fig. 1C). When patients were categorized according to gender, both women (1,731 ± 1,576) and men (1,742 ± 2.00) aged ≥42 years old, showed a higher expression of miR-200c-3p (*p*=0.003 and *p*=0.016, respectively) (Fig. 1D, 1E). Considering only the female sex, we found a higher expression of miR-200c-3p in group IV when compared to the other groups (181.4 ± 280.10, *p*=0.0002) (Fig. 1F). In males, we found a difference only between groups I and II when compared to group IV (0.810 ± 0.638, 1,299 ± 1,663 and 4,424 ± 7,373 respectively, *p*=0.046) (Fig. 1G).

When assessing the presence of coexisting comorbidities, concerning hypertension, we observed that group IV was the group that had the highest percentage of individuals with systemic arterial hypertension (SAH) 71.43% (n=10), *p*<0.0001 (Table 1). Similarly, group IV also had a higher percentage of patients with diabetes 42.86% (n=6) (*p*<0.0001). Interestingly, only patients belonging to groups III and IV had both hypertension and diabetes, and again, group IV has a higher percentage of patients with both comorbidities, being statistically significant when compared to the other groups 35.71% (n=5), *p*<0.0001. A logistic regression analysis was performed to identify variables independently associated with COVID-19. In this result, we found that the expression of miR-200c-3p and SAH are factors independently associated with patients with severe COVID-19 (Group IV), *p*<0.0001 (Table 1).

## DISCUSSION

Considering that there is still no specific treatment for COVID-19, the identification of molecules that can contribute to better patient management can be a useful tool in facing the pandemic [16]. MicroRNAs are small non-coding protein RNAs that have regulatory action and can serve as diagnostic and prognostic tools and even as tools for molecular targeted therapies [17, 18]. In the present study, we observed that miR-200c-3p showed different expression with some characteristics, such as higher expression in patients with more serious symptoms of the infection. Similar to our study, Liu Q et al. (2017) [19] observed that miR-200c-3p was upregulated in infections caused by the H5N1 virus, which, similar to SARS-CoV-2, can cause important mortality rates by inducing respiratory distress syndrome.

Buggele et al. found that in influenza A, miR-200c-3p increases its level of expression progressively after infection [13]. We believe that a similar event may be occurring in the infection caused by SAR-CoV-2, as in our results, we found a higher expression of this miR in patients with severe symptoms.

Lu et al., demonstrated through a luciferase assay that miR-200c is a direct target of ACE2 in human cardiomyocyte cells and suggested that this miR could be used as a preventive treatment by decreasing the expression levels of ACE2 [20], which is known to be the virus gateway to the cell. In contradiction, our findings show that the increased expression of miR-200c-3p in saliva samples from infected and uninfected patients by COVID-19 is related to a worse clinical course of the disease. Presumably, we advocated that the use of miR-200c-3p as a form of treatment should be performed with caution and further studies are needed to understand this mechanism.

Advanced age seems to be one of the factors with the worst prognosis in COVID-19 [10]. When comparing the expression of miR-200c with the age of the patients, without taking into account the evolution of the disease and regardless of sex, we found significant differences in the group with a mean ≥42 years. We also observed that when the patients were classified according to sex, both men and women showed greater expression of miR-200c-3p in the group of older patients. Thus, we speculate that in older individuals, the SARS-CoV-2 virus can positively regulate miR-200c-3p, which promotes the worst evolution of the disease.

Gender disparity was already evident in COVID-19, where lethality has been more prominent in males (17.7%) versus females (10.7%) [21]. MicroRNAs can exhibit different expression patterns in both sexes, and several factors may be responsible for this, such as hormonal influences and the chromosome in which they are encoded [22]. Pontecorvi G et al. suggested a potential role of gender-associated microRNAs in the regulation of several factors that may contribute to different responses to the pathogenicity and lethality of COVID-19 in men and women. In contrast, from other studies, miR-200c-3p did not show any difference concerning sex. Considering only the group composed of males, it is evident that group IV stands out with the expression of miR-200c-3p, similar to what was observed in females.

Similar to other relevant data presented in the literature, pre-existing comorbidities such as diabetes and hypertension do seem to have an unfavorable role in patients who oppose COVID-19. Corroborating these data, our study demonstrates that a higher percentage of patients who had diabetes and hypertension were higher in the two groups that had unfavorable characteristics, group III and IV. Notably, after logistic regression analysis, it was confirmed that in severe cases, the expression of miR-200c-3p is independently associated with those patients with hypertension. The results found in our study show that miR-200c-3p has differential expression in patients with more aggressive COVID-19 conditions, as well as revealing important associations with age.

The use of saliva as a biomaterial for the diagnosis of COVID-19 has been widespread since April 2020 after its release by the FDA. However, the use of this fluid presents some biases pointed out by the scientific community, mainly due to the lack of standardization of protocols, such as optimization of collection, transport, storage of samples, and test methods. However, sensitivity for diagnosis from saliva can vary from 91 to 98% [23]. Although our work does not have a diagnostic nature, but rather a prognosis, we are concerned with detailing our methodology in a way that contributes scientifically to the standardization of the protocols, also enabling bases for the validation of the experiments. 

Another important point in the rain of information about the SARs-CoV-2 pandemic is the increase in the number of articles found on pre-publication platforms (pre-prints). Quick access to information brings knowledge that can positively change the clinical outcome of countless patients [24], however, many of these pre-prints are not even reviewed and rarely published [25]. Nevertheless, it is well known that in many countries, mainly in Brazil and the USA, information from social media and preprints were used by doctors and politicians to defend specific treatments, an attitude that brings with it several obstacles that prevent the realization of studies quality and most likely can lead to inappropriate drug use and a high potential for harm to patients [25].

 Finally, these results, unique in the literature, may contribute to the use of this microRNA as a possible prognostic marker in the screening of patients. This work opens perspectives for the use of these important molecules in clinical practice, since the quantification of miRNAs is similar to one of the diagnostic techniques of COVID-19, qPCR.

## Conflict of Interest:

The authors declare that they have no conflict of interests.

## Supplementary Materials



## References

[1] Bartel DP (2004). MicroRNAs: genomics, biogenesis, mechanism, and function. Cell.

[2] Cullen BR (2011). Viruses and microRNAs: RISCy interactions with serious consequences. Genes Dev.

[3] Majer A, Caligiuri KA, Gale KK, Niu Y, Phillipson CS, Booth TF, Booth SA (2017). Induction of Multiple miR-200/182 Members in the Brains of Mice Are Associated with Acute Herpes Simplex Virus 1 Encephalitis. PLoS One.

[4] Canatan D, De Sanctis V (2020). The impact of MicroRNAs (miRNAs) on the genotype of coronaviruses. Acta Biomed.

[5] Tahamtan A, Inchley CS, Marzban M, Tavakoli-Yaraki M, Teymoori-Rad M, Nakstad B, Salimi V (2016). The role of microRNAs in respiratory viral infection: friend or foe?. Rev Med Virol.

[6] Rakhmetullina A (2020). How miRNAs can protect humans from coronaviruses COVID-19, SARS-CoV, and MERS-CoV.

[7] Guterres A, de Azeredo Lima CH, Miranda RL, Gadelha MR (2020). What is the potential function of microRNAs as biomarkers and therapeutic targets in COVID-19?. Infect Genet Evol.

[8] Report CdC-S: World Health Organization.

[9] (2020). Coronavirus COVID-19 outbreak: Latest news, information and updates.

[10] Wu Z, McGoogan JM (2020). Characteristics of and Important Lessons From the Coronavirus Disease 2019 (COVID-19) Outbreak in China: Summary of a Report of 72 314 Cases From the Chinese Center for Disease Control and Prevention. JAMA.

[11] Zhou F, Yu T, Du R, Fan G, Liu Y, Liu Z, Xiang J, Wang Y, Song B, Gu X, Guan L, Wei Y, Li H, Wu X, Xu J, Tu S, Zhang Y, Chen H, Cao B (2020). Clinical course and risk factors for mortality of adult inpatients with COVID-19 in Wuhan, China: a retrospective cohort study. Lancet.

[12] Jin JM, Bai P, He W, Wu F, Liu XF, Han DM, Liu S, Yang JK (2020). Gender Differences in Patients With COVID-19: Focus on Severity and Mortality. Front Public Health.

[13] Buggele WA, Johnson KE, Horvath CM (2012). Influenza A virus infection of human respiratory cells induces primary microRNA expression. J Biol Chem.

[14] Momen-Heravi F, Trachtenberg AJ, Kuo WP, Cheng YS (2014). Genomewide Study of Salivary MicroRNAs for Detection of Oral Cancer. J Dent Res.

[15] Livak KJ, Schmittgen TD (2001). Analysis of relative gene expression data using real-time quantitative PCR and the 2(-Delta Delta C(T)) Method. Methods.

[16] Viana N, Pimenta R, Gonçalves GL, Gomes TF, Guimaraes VR, Sro V, Sro M, Leite K, Reis S (2020). Hydroxychloroquine Efficacy for the Treatment of Patients with COVID-19: A Systematic Review and Meta-Analysis.

[17] Schwarz DS, Hutvagner G, Du T, Xu Z, Aronin N, Zamore PD (2003). Asymmetry in the assembly of the RNAi enzyme complex. Cell.

[18] Kim VN (2005). MicroRNA biogenesis: coordinated cropping and dicing. Nat Rev Mol Cell Biol..

[19] Liu Q, Du J, Yu X, Xu J, Huang F, Li X, Zhang C, Li X, Chang J, Shang D, Zhao Y, Tian M, Lu H, Xu J, Li C, Zhu H, Jin N, Jiang C (2017). miRNA-200c-3p is crucial in acute respiratory distress syndrome. Cell Discov.

[20] Lu D, Chatterjee S, Xiao K, Riedel I, Wang Y, Foo R, Bar C, Thum T (2020). MicroRNAs targeting the SARS-CoV-2 entry receptor ACE2 in cardiomyocytes. J Mol Cell Cardiol.

[21] EPIDEMIA COVID-19.

[22] Pontecorvi G, Bellenghi M, Ortona E, Carè A (2020). microRNAs as new possible actors in gender disparities of Covid-19 pandemic. Acta Physiol (Oxf).

[23] Czumbel LM, Kiss S, Farkas N, Mandel I, Hegyi A, Nagy Á, Lohinai Z, Szakacs Z, Hegyi P, Steward MC, Varga G (2020). Saliva as a Candidate for COVID-19 Diagnostic Testing: A Meta-Analysis. Front Med (Lausanne).

[24] Flanagin A, Fontanarosa PB, Bauchner H (2020). Preprints Involving Medical Research-Do the Benefits Outweigh the Challenges?. JAMA.

[25] Orsi C: Na pandemia, pré-prints servem mais à retórica que ciência.

