# Supplementary material for: MiR-200c-3p expression may be associated with worsening of the clinical course of patients with COVID-19
Source: Mol Biol Res Commun. 2021 Sep;10(3):141–7. doi: 10.22099/mbrc.2021.40555.1631 (PMC8340313; doi:10.22099/mbrc.2021.40555.1631)
Supplement: Supplementary file 1 [file mbrc-10-141-s001.pdf]

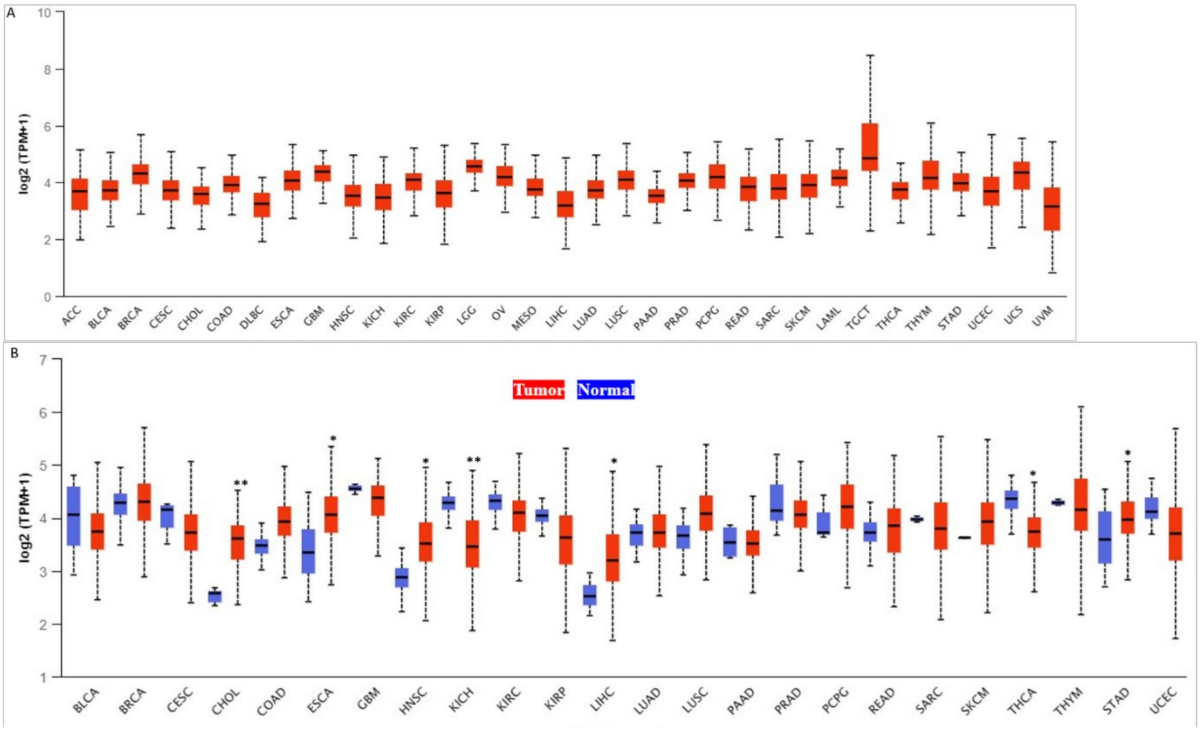

Figure S1: TRF1 expression in cancer. In A we showed the gene expression level of TRF1 in each TCGA cancer. In B we showed the comparison in TRF1 expression between normal and cancer tissues. The boxplots are grouped in pairs for each cancer, the control tissue is blue(left) and the tumor tissue in red(right). ** Log_2FC Cutoff: 1.0 q<0. 01. * Log_2FC Cutoff: 0.5 q<0.01.

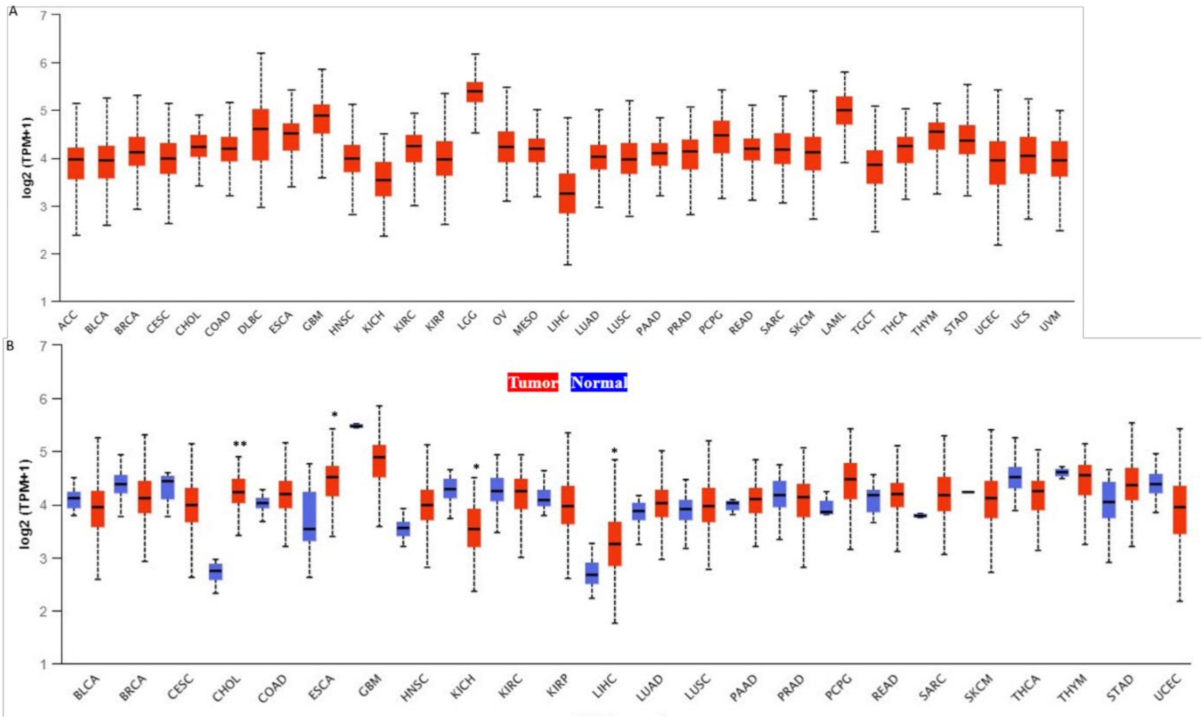

Figure S2: TRF2 expression in cancer. In A we showed the gene expression level of TRF2 in each TCGA cancer. In B we showed the comparison in TRF2 expression between normal and cancer tissues. The boxplots are grouped in pairs for each cancer, the control tissue is blue(left) and the tumor tissue in red(right). ** Log_2FC Cutoff: 1.0 q<0. 01. * Log_2FC Cutoff: 0.5 q<0.01.
